# The relative withdrawal of GFAP—An essential component of brain evolution

**DOI:** 10.3389/fnana.2025.1607603

**Published:** 2025-07-17

**Authors:** Mihály Kálmán

**Affiliations:** Department of Anatomy, Histology and Embryology, Semmelweis University, Budapest, Hungary

**Keywords:** lack of GFAP, birds, crocodilians, turtles, lizards, snakes, ray-finned fishes, cartilaginous fishes

## Abstract

The glial fibrillary acidic protein (GFAP) is the principal intermediate filament protein and histochemical marker for astroglia. It appears contradictory that there are extended GFAP-poor or even GFAP-free areas in the brains of various vertebrate clades: cartilaginous and ray-finned fishes, and amniotes. The “Relevant Subsections: Extended GFAP-free areas in various vertebrates” section in this study reviews our GFAP mapping studies on the brains of 58 species within these clades, as well as mappings from other authors, and demonstrates that these areas appeared independently from one another in the more advanced groups of different clades; it raises the supposition that the lack of GFAP is an apomorphic phenomenon. The GFAP expression has withdrawn mainly relatively: the GFAP-immunonegative areas increased more than the immunopositive ones. Primarily, regions that expanded and increased in complexity during evolution lack GFAP immunopositivity (except for their perivascular glia). The absence of GFAP expression, however, does not indicate the lack of astroglia. In the areas immunonegative to GFAP, astrocytes were visualized using other markers, such as glutamine synthetase or S-100 protein. In birds and mammals, lesions induced GFAP expression in these areas. It shows that the ability to express GFAP is not lost but has become facultative. These data suggest that the lack of GFAP production may provide an evolutionary advantage. The “Discussion” section relates the GFAP “withdrawal” to other steps of evolution: the increasing complexity and thickening of the brain wall, as well as the appearance of the astrocytes, particularly protoplasmic astrocytes, and then examines the proposed evolutionary advantages and disadvantages of the absence of GFAP. The role of the relative “withdrawal” of GFAP expression in brain evolution remains to be definitively answered. The most probable candidates may include the absence of synthesizing an unnecessary protein, improved adaptation of astrocytes to the demands of neurons, and an increased capacity for synaptic plasticity. In contrast, one must consider that the withdrawal of GFAP may not be a primary phenomenon but rather a consequence of the evolution of neural networks.

## 1 Introduction

The glial fibrillary acidic protein (GFAP) serves as the primary intermediate filament protein and histochemical marker of astroglia. It provides firmness to their processes and maintains their shape. Cell motility, maintenance of the blood–brain barrier (BBB), glial scar formation, response to hypoosmotic stress, anchoring of the cell membrane, and intracellular trafficking are also affected (Li et al., 2020; Messing and Brenner, 2020; Potokar et al., 2020). Early studies (Dahl and Bignami, 1973; Onteniente et al., 1983; Dahl et al., 1985) demonstrated that GFAP is present in the brains of various vertebrate classes, including cartilaginous and ray-finned fishes, reptiles, birds, and mammals. Furthermore, the antibodies raised against mammalian GFAPs react with the GFAPs of other vertebrate species. These studies, however, only demonstrated the presence of GFAP in representative species but did not map the distribution of immunopositive and -negative areas.

Since GFAP is the primary intermediate filament of ubiquity astroglia, it could be expected that every area is GFAP-immunopositive throughout any vertebrate brain. The Section 2 in this study reviews papers which demonstrate that surprisingly large brain areas poor in or even devoid of GFAP appeared in different vertebrate clades during evolution. The Section 3 examines the possible role of the absence of GFAP in brain evolution.

**Figure 1:**
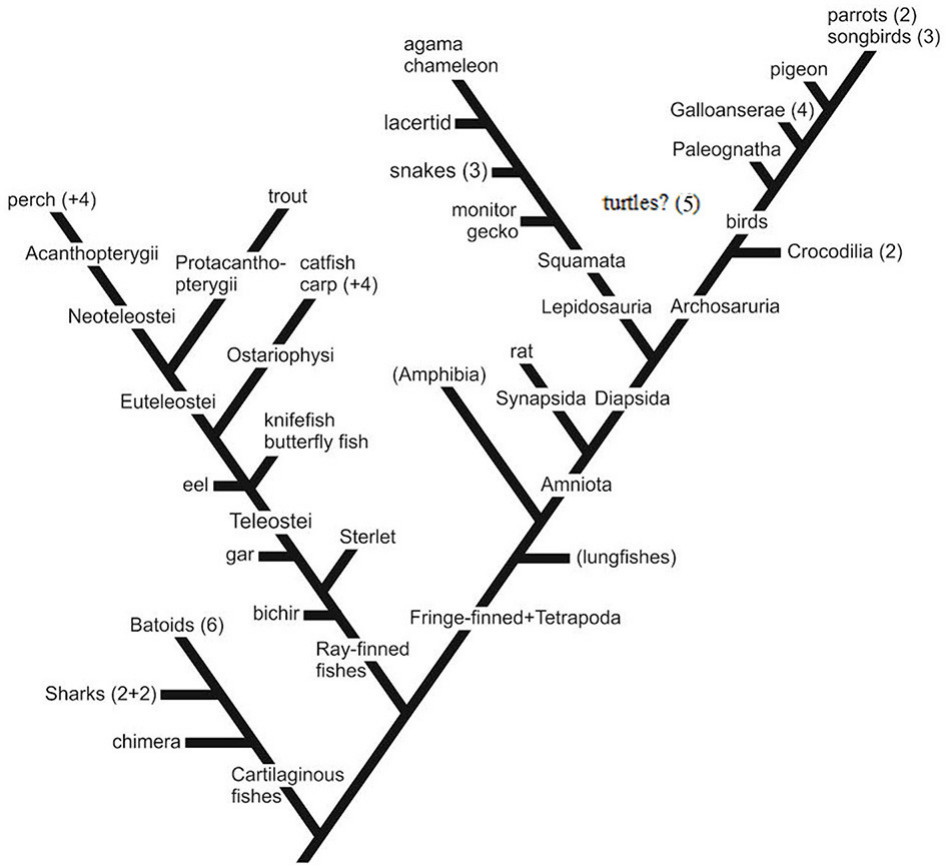
The phylogenetic positions of the species investigated. Simplified, only the colloquial names of the representative species of the groups investigated are applied. For the scientific names, see Tables 1–4. Non-represented groups are not shown, except for a few important ones in brackets (e.g., Paleognatha: ostrich and relatives). Numbers refer to the number of species from the group. Galloanserae (4), chicken-like (here: chicken+2 quail species) and goose-like (here: duck); Squamata comprises snakes and lizards, 3 and 5 species; batoids: skates and rays, eight species of the 3 orders; sharks: two squalomorph and two galeomorph species; perch+4: sander, two cichlids, sunfish; carp+4: goldfish, crucian carp, bram, blake; parrots (2): budgerigar and cockatiel; songbirds (3): two finches and magpie; eel, butterfly fish, and knifefish together mentioned in the text as “basal teleosts,” turtles (five species of fivefamilies): their position is unsure: lepidosaurs or archosaurs (Joyce, 2015; Lyson and Bever, 2020).

To enhance the readability of this article, the scientific names of the 58 species investigated in our study are not included in the text; rather, they are listed in Tables 1–4. The majority the names of species investigated by others can be found in the titles of the referenced articles (see References); if they are absent from these titles, they are mentioned within the body of our article. Not every study employing GFAP immunohistochemistry is referred to; only those that illustrate the distribution of immunopositivity are included. For orientation, a practically simplified “family tree” is presented in Figure 1.

**Table 1:** Birds investigated by Kálmán and Sebők (2023).

**Order/ suborder**	**Family/ subfamily**	**Species**
Galliformes^*^	Phasianidae/ Gallini	Domestic chicken, *Gallus gallus domesticus* LINNEAUS 1758
	Phasianidae/ Tetraogallini	King quail, Excalfactoria chinensis LINNEAUS 1766
		Japanese quail, *Coturnix japonica* TEMMINC and SCHLEGEL 1849
Anseriformes^*^	Anatidae	Muscovy duck, *Cairina moschata domestica* FLEMING 1922
Columbiformes^#^	Columbidae	Domestic pigeon, *Columba livia domestica* GMELIN 1789
Passeriformes/ Passeri^##^	Estrilidae	Zebra finch, *Taeniopygia guttata* VIEILLOT 1817
		Gouldian finch, *Erythrura gouldiae* GOULD, 1844
	Corvidae	Eurasian magpie, *Pica pica* LINNEAUS 1758
Psittaciformes^##^	Psittacidae	Budgerigar, *Melopsittacus undulatus* SHAW 1805
	Cacatuidae	Cockatiel, *Nymphicus hollandicus* KERR 1792

Every species was represented by two animals. Only taxa demonstrating the main phylogenetic relationships are given.

^*^They are of Galloanserae, opposite to Neoaves, which are divided into #Columbea and ^##^Passerea, according to Jarvis et al. (2014), Houde et al. (2019), and Braun and Kimball (2021). All species are of Neognatha. The Paleognatha (Struthioformes and Tinamusformes) were not represented.

## 2 Relevant subsections: extended GFAP-free areas in various vertebrates

### 2.1 Mammals and birds

**Figure 2:**
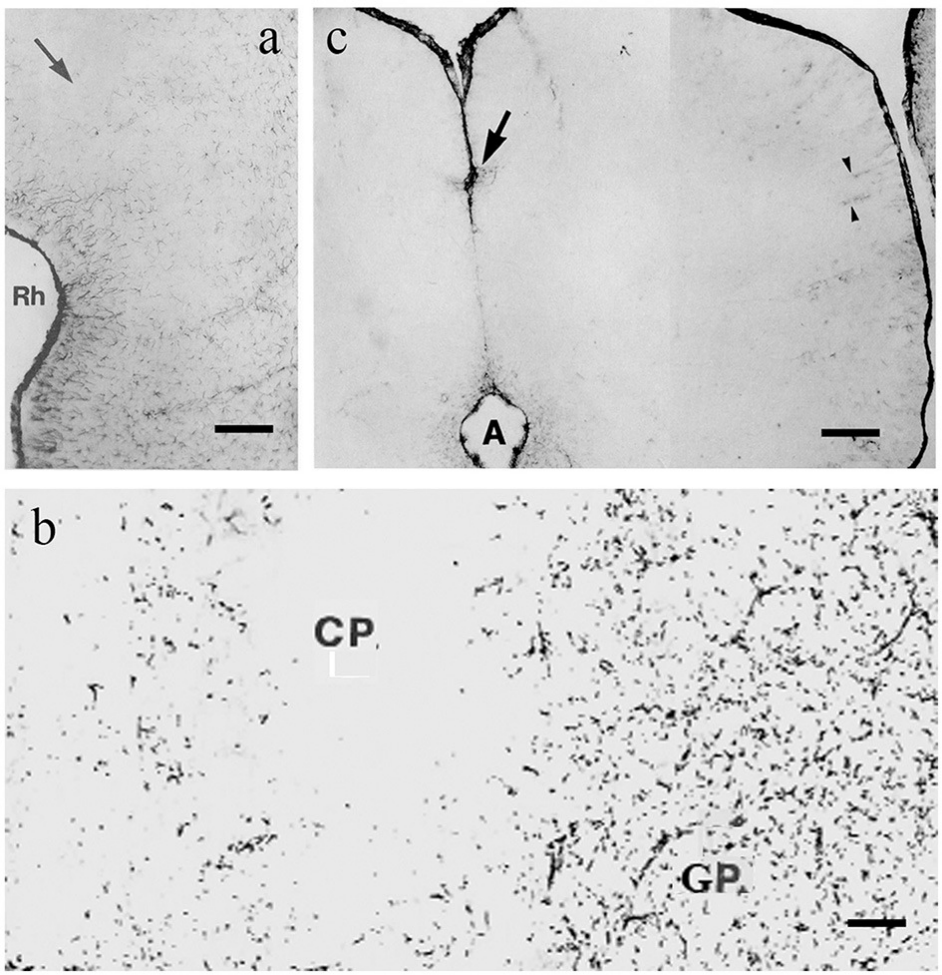
GFAP immunopositivity in rat; adapted from Kálmán and Hajós (1989). **(a)** Cortex, the midpart (arrow) is almost free of GFAP, it ceases below the rhinal sulcus (Rh); **(b)** Caudate-putamen (CP), almost free of GFAP-immunopositivity, and globus pallidus (GP), which is rich in it; **(c)** colliculus inferior, free of GFAP, except for a few cells around the cerebral aqueduct (A), at the arrow, and around vessels (arrowheads). Scale bars: **(a)** 200 μm; **(b)** 80 μm; and **(c)** 70 μm.

The most extensive GFAP-free or -poor areas are found in these groups, which are predominant in the forebrain and midbrain. In rat, as the representatives of mammals, a wide middle zone of neocortex (dorsal cortex), approximately the layers 2–4, is very poor in GFAP, almost free of it (Ludwin et al., 1976; Kálmán and Hajós, 1989; Zilles et al., 1991; Figure 2a). These layers do not exist in reptiles; they are new acquisitions in evolution (Reiner et al., 1984; Briscoe and Ragsdale, 2018). However, in the paleocortex (lateral cortex), there is no similar GFAP-free zone even in rats (Kálmán and Hajós, 1989; Zilles et al., 1991; Figure 2a). The striatum (caudate-putamen, Figure 2b) and the thalamic nuclei, except for the nucleus reticularis, are also very poor in GFAP in rats (Kálmán and Hajós, 1989; Zilles et al., 1991). The tectum is almost entirely GFAP-free (Hajós and Kálmán, 1989; Zilles et al., 1991; Figure 2c). Biochemical methods have also demonstrated the uneven distribution of GFAP (Patel et al., 1985).

Besides rats, there is only one mapping study, which was found on a mammal, on a shrew (Olkowicz et al., 2004). It revealed extended GFAP-free areas similar to those found in rats. Colombo et al. (2000) and Falcone et al. (2019) studied several mammalian species, focusing on the cortices and specifically examining the interlaminar astrocyte processes. In contrast, Falcone et al. (2021) concentrated on the so-called varicose-projection astrocytes (VP-As) cells. The other mammalian studies focus only on confined areas, rather than mapping the whole brain.

**Figure 3:**
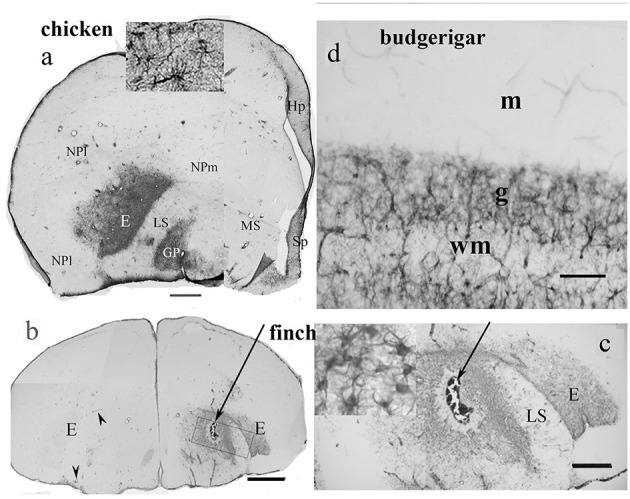
GFAP distribution in bird telencephalon; adapted from Kálmán and Sebők (2023). **(a)** Domestic chicken. In the entopallium (E) and the globus pallidus (GP), there is a dense population of GFAP-immunopositive astrocytes (see enlarged in the inset). Hp, hippocampus; HP, hyperpallium; LS, lateral striatum; MP, mesopallium; NPl and m, nidopallium laterale and mediale; Sp, septum. Scale bar: 1.4 mm **(b)** and **(c)** Gouldian finch. The intact left-side entopallium (E) is devoid of GFAP-immunopositive astrocytes, except for the perivascular astrocytes, which are GFAP-immunopositive. Following the lesion (arrow, right hemisphere) the astrocytes of the entopallium became GFAP-immunopositive. The perivascular astrocytes are GFAP-immunopositive in the otherwise immunonegative intact areas. Inset in **(c)** shows the astrocytes enlarged. Scale bars: **(b)** 1.4 mm; **(c)** 250 μm. **(d)** The other GFAP-immunopositive areas were similar in every species (here: budgerigar cerebellum). The Bergmann glia contains no GFAP but vimentin in either species; g, m: granular and molecular layers; wm: white matter. Scale bar: 70 μm.

In birds, according to our studies on chicken (Kálmán et al., 1993, 1998) the hyperpallium, mesopallium, and nidopallium proved to be GFAP immunonegatíve (Figure 3a, note, the terminology of avian neuroanatomy has been reformed by Reiner et al., 2004) as well as the striatum, the upper layers of tectum, several thalamic nuclei, and the molecular layer of cerebellum. Cameron-Curry et al. (1991) had published similar results in quail. The upper layers of chicken tectum were also found to be devoid of GFAP by Linser (1985). Vimentin, an intermediate filament protein characteristic of the immature glia (Dahl, 1981), substitutes for GFAP in the molecular layer of the cerebellum (Roeling and Feirabend, 1988; Kálmán et al., 1998) but not in the other GFAP-free areas, either in mammals or birds (Kálmán et al., 1998).

Our recent study (Kálmán and Sebők, 2023) compared the GFAP distribution in 10 avian species (Table 1, Figure 1), four of Galloanserae (duck, chicken, and two quail species), one of “older” Neoaves (Columbea, pigeon), and five of “newer” Neoaves (Passerea, two parrots, and three songbirds: two finch species and magpie). The phylogenetic relations were based on the family trees published by Jarvis et al. (2014), Houde et al. (2019), and Braun and Kimball (2021). In all the species studied, the distribution of GFAP immunopositivity was similar to that found in chickens (Kálmán et al., 1993, 1998), except for the entopallium.

The entopallium, a visual center, was found to be GFAP-immunopositive (Figure 3a) in the representatives of phylogenetically “older” groups (Galloanserae and Columbea), but not (Figure 3b) in the representatives of “younger” avian groups, such as songbirds and parrots. So, a territory lost its GFAP immunopositivity during the evolution of birds. In other brain areas, no alterations were found (Kálmán and Sebők, 2023). Brains of songbirds and parrots are typically considered the most advanced avian brains by the brain-to-body ratio (Jerison, 1973), neuron density, and task-solving capability (Wyles et al., 1983; Dicke and Roth, 2016; Olkowicz et al., 2016).

### 2.2 GFAP immunonegativity does not mean the lack of astroglia

Counting the astrocytes in semithin sections of rat brain, their number did not differ considerably in the GFAP-rich and GFAP-immunonegative brain areas (Hajós et al., 1993). A similar result was obtained with classical impregnation methods (Bailey and Shipley, 1993). Several studies (e.g., Connor and Berkowitz, 1985; Walz and Lang, 1998; Sofroniew and Vinters, 2010) mention that not all astrocytes produce GFAP, or at least not at a detectable level, or in depolymerized form, which is not detectable immunohistochemically (Hajós et al., 1993). In the absence of GFAP, astrocytes were detected using other immunohistochemical reactions, most frequently against glutamine synthetase or S-100 protein (Ludwin et al., 1976; Norenberg and Martinez-Hernandez, 1979, rat; Linser, 1985, chicken).

Both in mammals and birds, an intense GFAP immunoreactivity appears following injury, even in those areas which are devoid of GFAP in intact animals (Bignami and Dahl, 1976); it was also observed in the resident glia (Ajtai and Kálmán, 1998, Bergmann glia in chicken), not only in the reactive glia. Other stimuli can also induce GFAP production, for example, a blockade of afferent activity in the chicken cochlear nucleus (Canady and Rubel, 1992). These results demonstrate that the lack of GFAP expression is not due to incapability but a reversible inactivation, such as suppression. The GFAP expression has become facultative in these areas. In a population of GFAP-immunonegative astrocytes, GFAP mRNA was detected, indicating that synthesis was blocked at the translation level (Zhou et al., 2000).

It is noteworthy that the perivascular glia proved to be GFAP-immunopositive even in areas otherwise free of GFAP (Kálmán and Hajós, 1989; Zilles et al., 1991; Kálmán et al., 1993; Kálmán and Sebők, 2023), see also Figures 2c, 3. This suggests that the lack of GFAP immunostaining is not due to histotechnical faults.

Finally, our current understanding of GFAP distribution may be revised after extensive studies on GFAP isoforms, including GFAP δ, κ, and others (Holy and Pekny, 2015; Falcone, 2022).

### 2.3 No areas are devoid of GFAP in turtles and crocodilians

**Figure 4:**
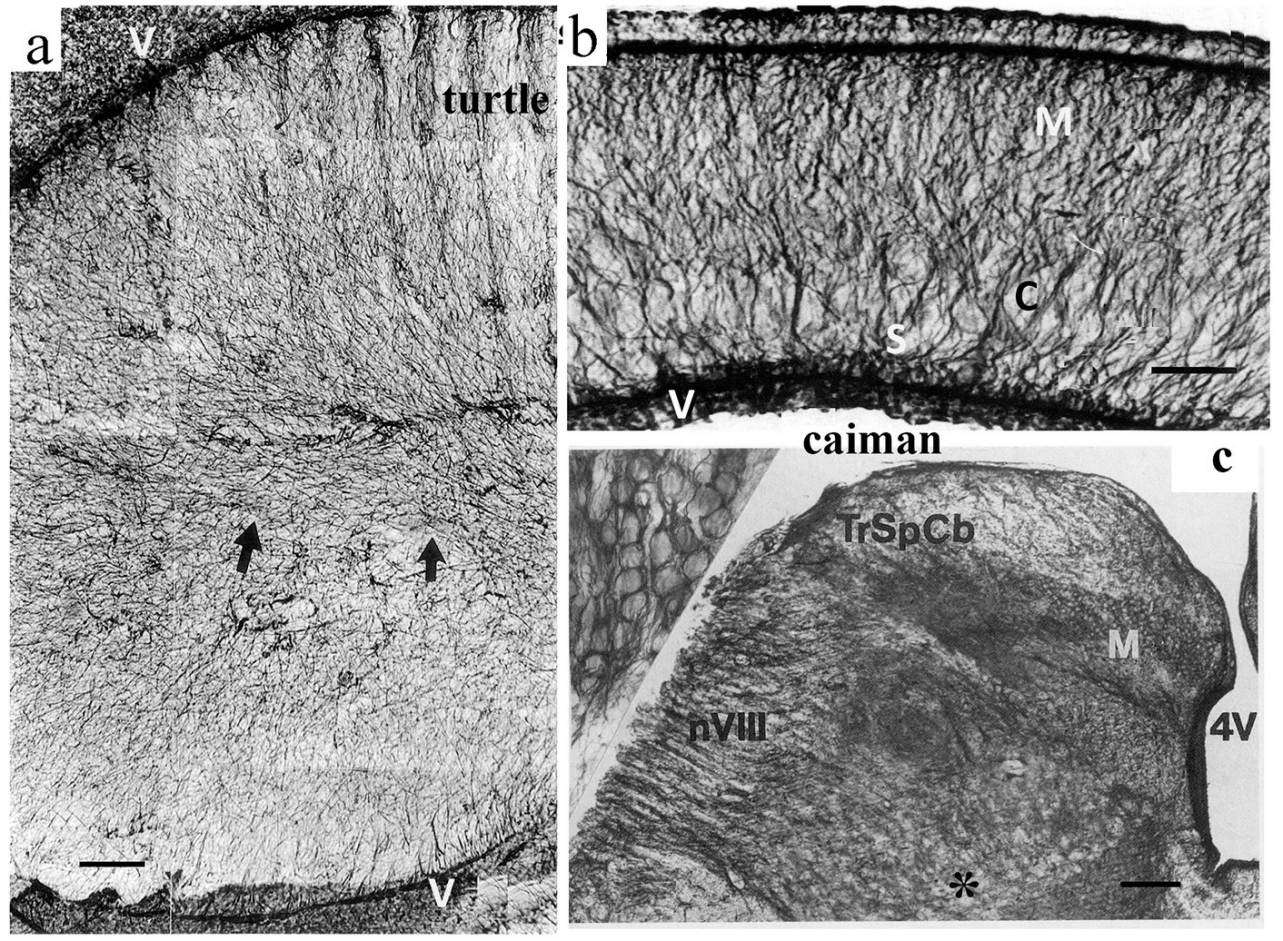
GFAP immunopositivity in details of the turtle and caiman brains. **(a)** Turtle DVR (dorsal ventricular ridge), full of GFAP-immunopositive elements; adapted from Kálmán et al. (1994). Compare it to the nidopallium and mesopallium of the chicken in Figure 3. Arrows point to the central glial bundle. Note that the GFAP-immunopositive glial cells are represented here by long, thin, fiber-like cells: tanycytes (Horstmann, 1954). V: Surface of the lateral ventricle. Scale bar: 100 μm. **(b)** Caiman dorsal cortex; adapted from Kálmán and Pritz (2001). Note the evenly dense immunopositivity. C: cell-rich layer; M: motoric axon layer; S: sensory axon layer; V: Surface of the lateral ventricle. Scale bar, 150 μm. **(c)** The posterior border of the fourth ventricle (4V) in caiman. M: Nucleus magnocellularis cochlearis, in its enlarged detail (inset, left upper corner), the perineuronal glial rings are well visible; nVIII: root of the vestibulocochlear nerve; TrSpCb: Tractus spinocerebellaris dors. Scale: 250 μm, for the inset: 25 μm.

**Figure 5:**
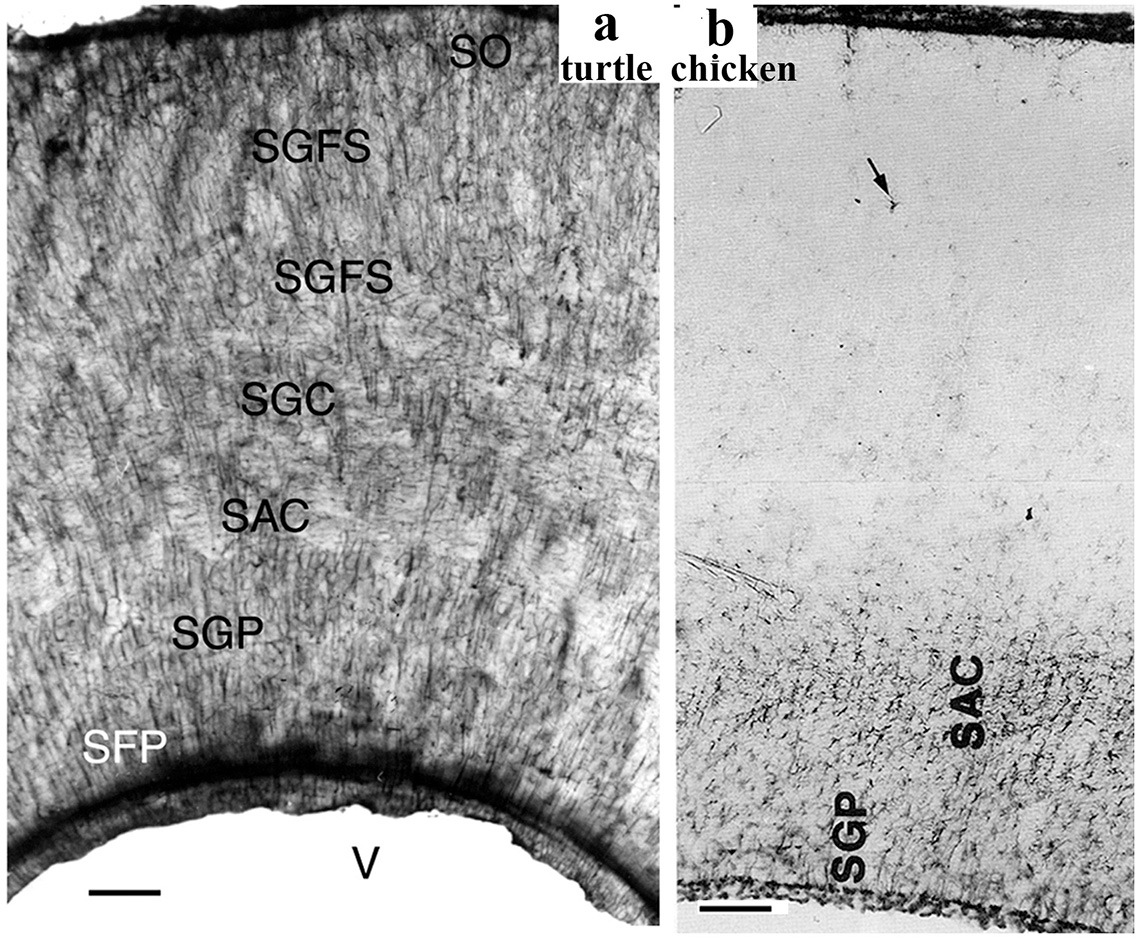
GFAP immunopositivity in the turtle and chicken tecta. **(a)** Turtle tectum, a detail of the wall around the tectal ventricle (V); adapted from Kálmán et al. (1994). All the layers are immunopositive (SFP, stratum fibrosum periventriculare; SGP, stratum griseum periventriculare; SAC and SGC, stratum album and griseum centrale; SGFS, stratum griseumfibrosum superficiale; SO, stratum opticum; note: griseum: gray; album: white). Scale bar: 80 μm. **(b)** A similar detail from chicken; adapted from Kálmán et al. (1993). GFAP immunopositivity is confined to the deep layers. Above them, astrocytes are very infrequent (arrow). Scale bar: 100 μm.

Neither turtles nor crocodilians had GFAP-free areas comparable to those found in birds (Kálmán et al., 1994, 1997; Kálmán and Pritz, 2001; Lazzari and Franceschini, 2006; Lőrincz and Kálmán, 2020a,b, Table 2). The areas homologous with the GFAP-free areas of birds are densely GFAP-immunopositive in turtles and crocodilians: the dorsal pallium (Figure 4a), and dorsal ventricular ridge (DVR, Figure 4b), as well as the striatum, the superior layers of tectum (Figure 5), several thalamic nuclei, and the molecular layer of cerebellum. Perineuronal glial rings in the nucleus magnocellularis cochlearis were detected by GFAP immunostaining in caiman (Kálmán and Pritz, 2001) but not in adult chicken (Kálmán et al., 1993, 1998) (Figure 4c).

**Table 2:** Reptiles in our studies.

**Order (subclass)**	**Suborder**	**Family**	**Species and the number of animals**
^#^Testudines (undefined)	Cryptodira	Testudinidae	Greek tortoise, *Testudo hermanni boettgeri*, MOJSISOVICS 1889 (1)
		Emydidae	^*^Red-eared slider, *Trachemys scripta elegans*, WIED 1838 (2)
		Geoemydidae	Chinese stripe-necked turtle, *Mauremys sinensis*, GRAY 1834 (2)
			^**^Spanish pond turtle *Mauremys leprosa* SCHWEIGGER 1812 (1)
	Pleurodira	Pelomedusidae	African helmeted turtle, *Pelomedusa subrufa*, BONNATERRE 1789 (2)
Crocodilia (Archosauria)		Alligatoridae	^***^ Spectacled caiman, *Caiman crocodilus* LINNEAUS 1758 (2)
			^****^Cuvier's dwarf caiman *Paleosuchus palpebrosus*, CUVIER 1807 (2)
Squamata, lizards, and snakes (Lepidosauria)	Gekkota	Eublepharidae	Leopard gecko, *Eublepharis macularius*, BLYTH 1854 (4)
	Lacertomorpha	Lacertidae	Moroccan eyed lizard, *Timon tangitanus*, BOULENGER 1889 (2)
	Anguimorpha	Varanidae	Savannah monitor, *Varanus exanthematicus*, BOSC 1792 *(*1)
	Serpentes	Boidae	Columbian rainbow boa, *Epicrates cenchria maura*, LINNAEUS 1758 (2)
		Pythonidae	Ball python. *Python regius*, SHAW 1802 (2)
		Colubridae	Corn snake, *Pantherophis guttatus*, LINNAEUS 1766 (3)
	Iguania	Agamidae	Bearded dragon, *Pogona vitticeps*, AHL, 1926 (4)
		Chamaeleonidae	Veiled chameleon, *Chamaeleo calyptratus*, DUMÉRIL & DUMÉRIL, 1851 (3)

Only taxa demonstrating the main phylogenetic relationships are given according to Wiens et al. (2012) and Pyron et al. (2013). Numbers in brackets: the number of animals.

Crocodilia belong to the Archosauria together with the birds and extinct groups (dinosaurs, pterosaurs).

^#^The position of turtles has not been definite. Formerly, they were positioned as Anapsida (Carroll, 1988; Prothero, 2007). Presently they are held diapsids (Figure 1), but it has not been definite whether archosaurs, lepidosaurs, or they are separate from both as a sister group of all diapsids (Joyce, 2015; Lyson and Bever, 2020).

^*^Kálmán et al. (1994); ^**^ Kálmán et al. (1997); ^***^ Kálmán and Pritz (2001); ^****^Lőrincz and Kálmán (2020b); unmarked: Lőrincz and Kálmán (2020a).

Formerly, held as anapsid reptiles (Carroll, 1988; Prothero, 2007), and the closest extant relatives of stock-reptiles; recently, they are regarded as diapsids. Within this group, whether they are sister groups of lepidosaurs, or archosaurs, or all of the diapsids, the relationship remains indefinite, and it is beyond the scope of our study; see, for example, the studies by Joyce (2015) and Lyson and Bever (2020). In either case, the turtles are usually held as the most ancestral branch of extant reptiles. Our groups investigated five species (Kálmán et al., 1994, 1997; Lőrincz and Kálmán, 2020a) representing different families, including one representative of Pleurodira; a sixth species was described by Lazzari and Franceschini (2006). Crocodilians are the closest extant relatives of birds (Wheatstone and Martin, 1979). Two species were investigated (Kálmán and Pritz, 2001; Lőrincz and Kálmán, 2020b).

Our studies suggest that the GFAP-immunonegative areas may be characteristic of more advanced brains (avian and mammalian species), whereas the homologous areas in turtles and crocodilians are rich in GFAP. Therefore, the GFAP-immunonegative areas may be regarded as advanced, apomorphic features, which have developed independently in mammals and birds, as they are not present in either turtles or crocodiles. The GFAP-immunonegative areas increased more than the immunopositive ones; therefore, the withdrawal of the latter ones was relative. It appears that those areas are GFAP-immunonegative in mammals and birds, which have undergone enlargement and increased complexity during evolution, as if the “new” components had been built in without the presence of GFAP.

On the other hand, in some glial populations (e.g., the Bergmann glia and the perineuronal rings in the cochlear nuclei), it can be seen that they are GFAP-immunopositive in a crocodilian (i.e., caiman, Kálmán and Pritz, 2001). Still, they are not immunopositive in birds (i.e., chicken; Kálmán et al., 1993, 1998). However, their GFAP expression is only inactivated but appears following proper stimulation (Ajtai and Kálmán, 1998; Bergmann glia; Canady and Rubel, 1992; cochlear nucleus).

### 2.4 The other reptilian clade, Squamata (snakes and lizards), Lepidosauria

The Squamata (lizards and snakes) are lepidosaurs, which developed independently from birds and crocodilians, which belong to the archosaurs (Figure 1). The phylogenetic relationships between the Squamata species investigated are estimated according to Wiens et al. (2012) and Pyron et al. (2013). Squamata is one of the richest and most diverse extant vertebrate groups. The astroglial patterns mirror this diversity.

Our study (Lőrincz and Kálmán, 2020a) investigated the distribution of GFAP-immunopositive elements in five lizards and three snake species, each represented a different family (Table 2). The species investigated formed three groups according to the distribution of GFAP immunopositivity.

**Figure 6:**
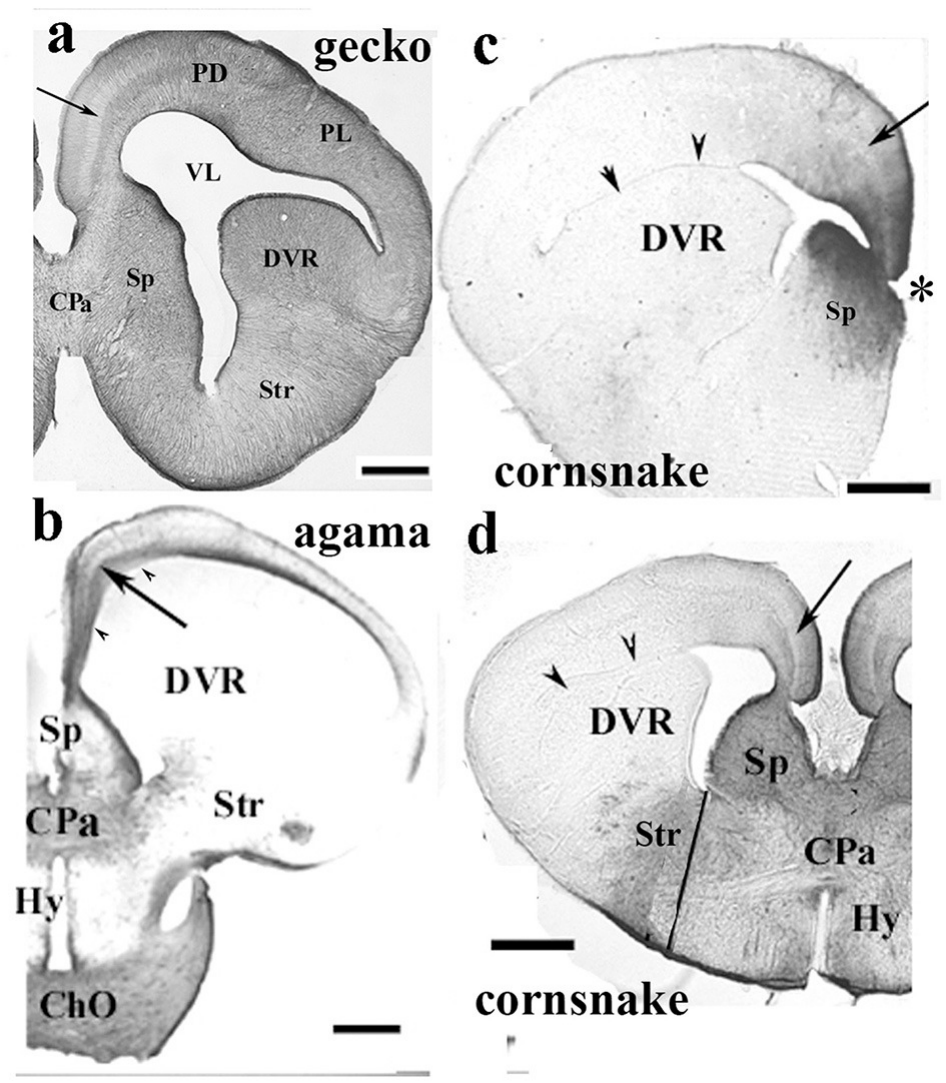
Various patterns of GFAP distribution in Squamata telencephala; adapted from Lőrincz and Kálmán (2020a). ChO, optic chiasma; Cpa, pallial commissure; DVR, dorsal ventricular ridge; Hy, hypothalamus; PD and PL, dorsal and lateral pallium; Sp, septum; Str, striatum; VL, lateral ventricle, where it is compressed: arrowheads. The light, GFAP-poor zone in the middle of the trilaminar pattern of the medial and mediodorsal pallia is shown with an arrow. Nissl counterstaining demonstrated (Lőrincz and Kálmán, 2020a) that this zone concentrates the neurons. **(a)** Gecko; a GFAP-rich lizard brain, a telencephalic section through the pallial commissure. Scale bar: 400 μm. **(b)** Agama; a GFAP-poor brain, a telencephalic section through the pallial commissure. The dorsal and lateral pallia, DVR, and hypothalamus remain almost free of GFAP. Scale bar: 600 μm. **(c)** Cornsnake; a telencephalic section at the interventricular foramen (asterisk), the territory of GFAP immunopositivity is confined to the medial and mediodorsal pallium, and the adjacent part of the septum. The trilaminar pattern is hardly recognizable (arrow). Scale bar: 800 μm. **(d)** Cornsake: a section at the pallial commissure. The line to the ventral sulcus of the DVR approximately separates the striatum and hypothalamus. Note that the GFAP-immunopositive area is much larger than in the rostral section in **(c)** and has an intermediate extension between the gecko and agama sectioned also at the pallial commissure. Scale bar: 800 μm.

**Figure 7:**
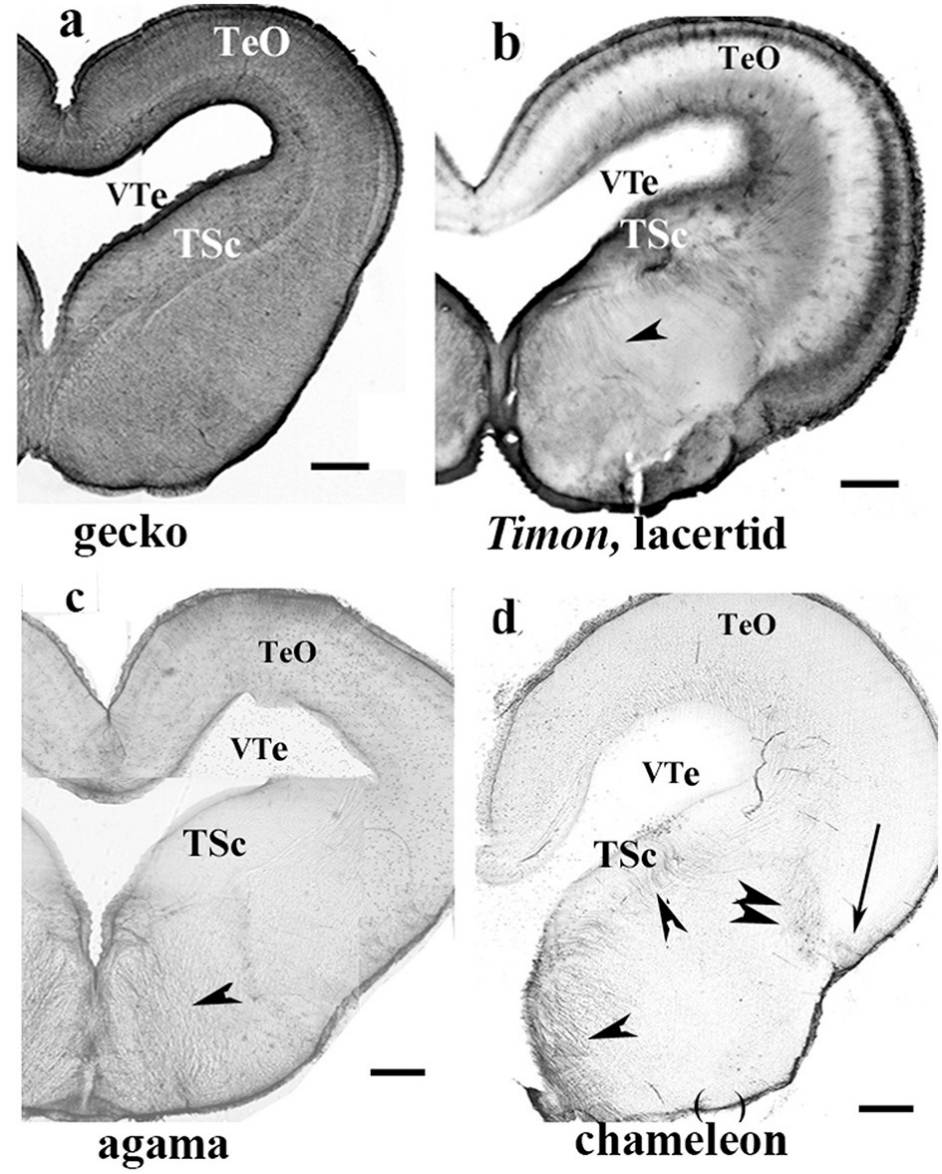
Various patterns of GFAP distribution in Squamata mesencephala; adapted from Lőrincz and Kálmán (2020a). TeO, optic tectum; TSc, torus semicircularis; VTe, tectal ventricle. **(a)** Gecko; both the tegmentum and tectum display a dense GFAP immunopositivity. Scale bar: 400 μm. **(b)** *Timon*, a lacertid lizard; its mesencephalon exhibits poor GFAP immunopositivity, with relatively more GFAP detected in the outer zone of the tectum and the tegmentum (arrowhead). The extension of GFAP immunopositivity quasi-represents an intermediate state between those of the gecko and agama, as shown in **(c)**. Scale bar: 320 μm. **(c)** Agama; the GFAP immunopositivity is confined to the superficial and deep layers in the tectum. The tegmentum has a loose system of radial glial processes (arrowhead). Scale bar: 500 μm. **(d)** Chameleon; its mesencephalon is very poor in GFAP-immunopositive structures, which are confined to a few groups of radial glia (arrow, arrowheads) and astrocytes (double arrowhead). Scale bar: 320 μm.

In the gecko (Figure 6a, see also Lazzari and Franceschini, 2005a), monitor lizard, as well as in *Anolis* (Lazzari and Franceschini, 2005b), the telencephalon, tectum, and all the other brain parts were rich in GFAP. At the other end, the agama (Figure 6b) and the chameleon were positioned. In their brains, the GFAP–containing territories were very confined (mainly to the septum, parts of the striatum). The lacertid lizard *Timon*, along with the snakes (a boa and a python species, and a cornsnake, Figures 6c, d), were in an intermediate position, as only the tectum and a portion of the DVR proved to be GFAP-immunopositive. In snakes, the distribution of GFAP was very similar in the three species representing three families. The most rostral and the dorsal parts of the telencephalon were free of GFAP (Figures 6c, d). The Squamata tecta also displayed a phylogenetic gradient of GFAP immunopositivity (Figure 7).

A narrow middle zone (Figures 6a–d) of medial and mediodorsal pallia was poor in GFAP in every Squamata species investigated, but not in turtles and crocodilians. In this zone, counterstaining according to Nissl revealed densely packed neurons. See also Font et al. (2001, *Tarentola mauretanica*, Gekkonidae), Lazzari and Franceschini (2001, 2005a), and Ahboucha et al. (2003, *Eumeces algeriensis*, Scincoidae; *Agama impalearis*, Agamidae; *Tarentola mauritanica*, Gekkonidae).

According to our results (Lőrincz and Kálmán, 2020a), geckos and monitor lizards, in which astroglia are GFAP-immunopositive in every brain area, similarly to that found in turtles, can be considered plesiomorphic. The geckos belong to a sister group (Gekkota) of all other lepidosaurs (Wiens et al., 2012; Pyron et al., 2013). In contrast, agama and chameleon, which have the most extensive GFAP-free areas, belong to Iguania, which is considered the most advanced group. From the clade of Squamata lacertids branched out first, then snakes (Wiens et al., 2012; Pyron et al., 2013; Figure 1). In these latter two groups, the extension of GFAP-immunopositive areas has intermediate positions between gecko and agama.

### 2.5 Ray-finned fishes

Following previous studies of carp (Kálmán, 1998), goldfish (Kálmán and Ajtai, 2000), and sterlet (Kálmán and Ari, 2002), we investigated (Kálmán et al., 2021, Table 3) three species as representatives of different non-teleost groups (bicirrh, sterlet, and gar), three species (eel, butterfly fish, knifefish) as representatives of the “basal” teleost groups, that is, which are ancestral to the division of Ostariophysi and Euteleostei. Of Ostariophysi carp and four species of its relatives (crucian carp, goldfish, bram, and blake), and one catfish were studied, and of Euteleostei six species (trout, perch, sander, lemon cichlid, angelfish, and sunfish). The phylogenetic relations are shown in Figure 1 after Betancur-R et al. (2013, 2017).

**Table 3:** Ray-finned fishes studied in Kálmán et al. (2021).

**Main groups**		**Order and category above it**	Family/subfamily^*^	**Species**
Non-Teleostei^*^		Cladistia/Polypteriformes	Polypteridae	Senegal bichir, *Polypterus senegalus*, CUVIER, 1829 (2)
		Chondrostei/Acipenseriformes	Acipenseridae	Sterlet, *Acipenser ruthenus*, LINNEAUS 1758 (2)#
		Ganoidei/Lepisosteiformes	Lepisosteidae	Gar, *Lepisosteus oculatus* WINCHELL 1864 (2)
“Basal”^**^ Teleostei		Elopomorpha/Anguilliformes	Anguillidae	European eel, *Anguilla anguilla*, LINNEAUS 1758 (2)
		Osteoglossomorpha Osteoglossiformes	Pantodontidae^@^	Freshwater butterfly fish, *Pantodon buchholzi*, PETERS 1877 (2)
			Notopteridae	Reticulate knifefish, *Papyrocranus afer* GÜNTHER 1968 (2)
Ostariophysi		Cypriniformes	Cyprinidae/Cyprinninae	Carp, *Cyprinus carpio* LINNEAUS 1758 (2)^##^
				Crucian carp, *Carassius carassius*. LINNEAUS 1758 (2)
				Goldfish. *Carassius auratus*, LINNEAUS 1758 (2)^###^
			Cyprinidae/Leuciscinae^@^	Bream. *Abramis brama*. LINNEAUS 1758 (2)
			Cyprinidae/Alburninae^@^	Common bleak. *Alburnus alburnus* LINNEAUS 1758 (2)
		Siluriformes	Icturidae	Brown catfish, *Icturus nebulosus* LESUEUR 1819 (2)
Euteleostei	Protacantho-pterygii	Salmoniformes	Salmonidae	Rainbow trout, *Onorhynchus mykiss* WALBAUM 1792 (3)
	Neoteleostei–Acantho-pterygii	Ovalenteria Cichliformes	Cichlidae	Ereshwater angelfish, P*terophyllum scalare* LICHTENSTEIN 1923 (1)
				Lemon cichlid, *Neolamprologus leleupi*, POLL 1956 (2)
		Percomorpharia Perciformes	Percidae	Perch, *Perca fluviatilis*, LINNEAUS 1758 (1)
				Pike-perch or sander. *Sander lucioperca*, LINNEAUS 1758 (1)
		Percomorpharia Centrarchiformes	Centrarchidae	Pumpkinseed or sunfish, *Lepomi*s *gibbosu*s, LINNEAUS s, 1758 (2)

On the basis of Betancur-R et al. (2013, 2017), modified. Only the taxa that demonstrate phylogenetic relationships are given. Numbers in brackets: the number of animals.

^*^Not an official term.

^**^The term “basal” is objected to by several authors, but no other common term has been found for the teleost groups positioned before the divergence of Ostaryophys and Euteleostei.

^@^Recently, in some systems, in a separate order, Pantodontiformes, and recently, in some systems, in a separate family, Leuciscidae.

^#^Also in Kálmán and Ari (2002).

^##^Also in Kálmán (1998).

^###^Also in Kálmán and Ajtai (2000).

**Figure 8:**
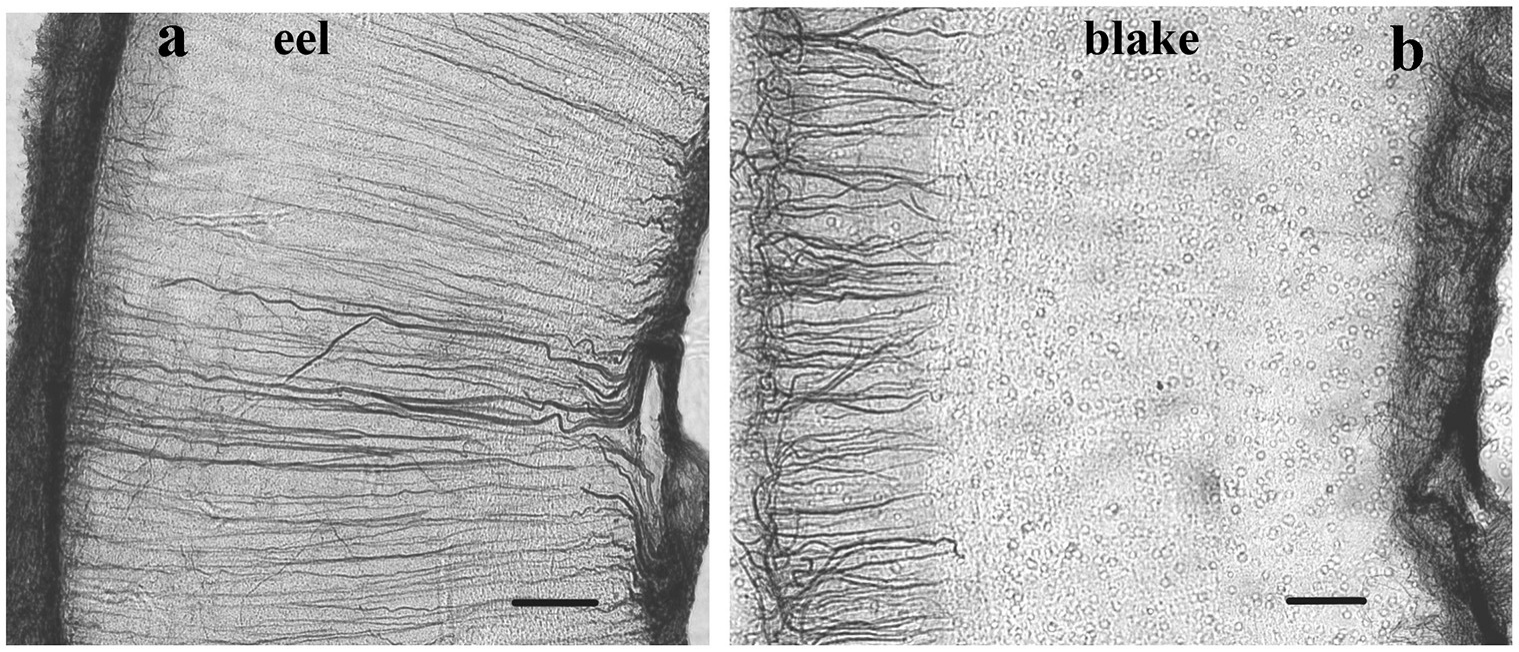
Ray-finned fishes, details of the tectal wall; adapted from Kálmán et al. (2021). The meningeal surface is on the left side. **(a)** A basal teleost (eel); the long, thin glial cells (tanycytes) are GFAP-immunostained in full length. **(b)** A representative of Ostariophysi (blake), the tanycytes are visualized well only in the upper (submeningeal, left side) part of the section. Scale bars: 80 μm.

GFAP-free areas included the molecular layer of cerebellum in Cyprinidae and the deeper layers of the tectum in Ostariophysi and Euteleostei (Figure 8). Note that Meek (1983) demonstrated using impregnation methods, that in their tectum, the glial processes also originate from the ependyma; however, the periventricular part is thin and poor intermediate filaments, as observed by electron microscopic observation of Stevenson and Yoon (1982). Mass of optic tectum and cerebellum relatively increased in these groups as compared to the non-teleosts and “basal teleosts” (Cerda-Reverter et al., 2008).

**Figure 9:**
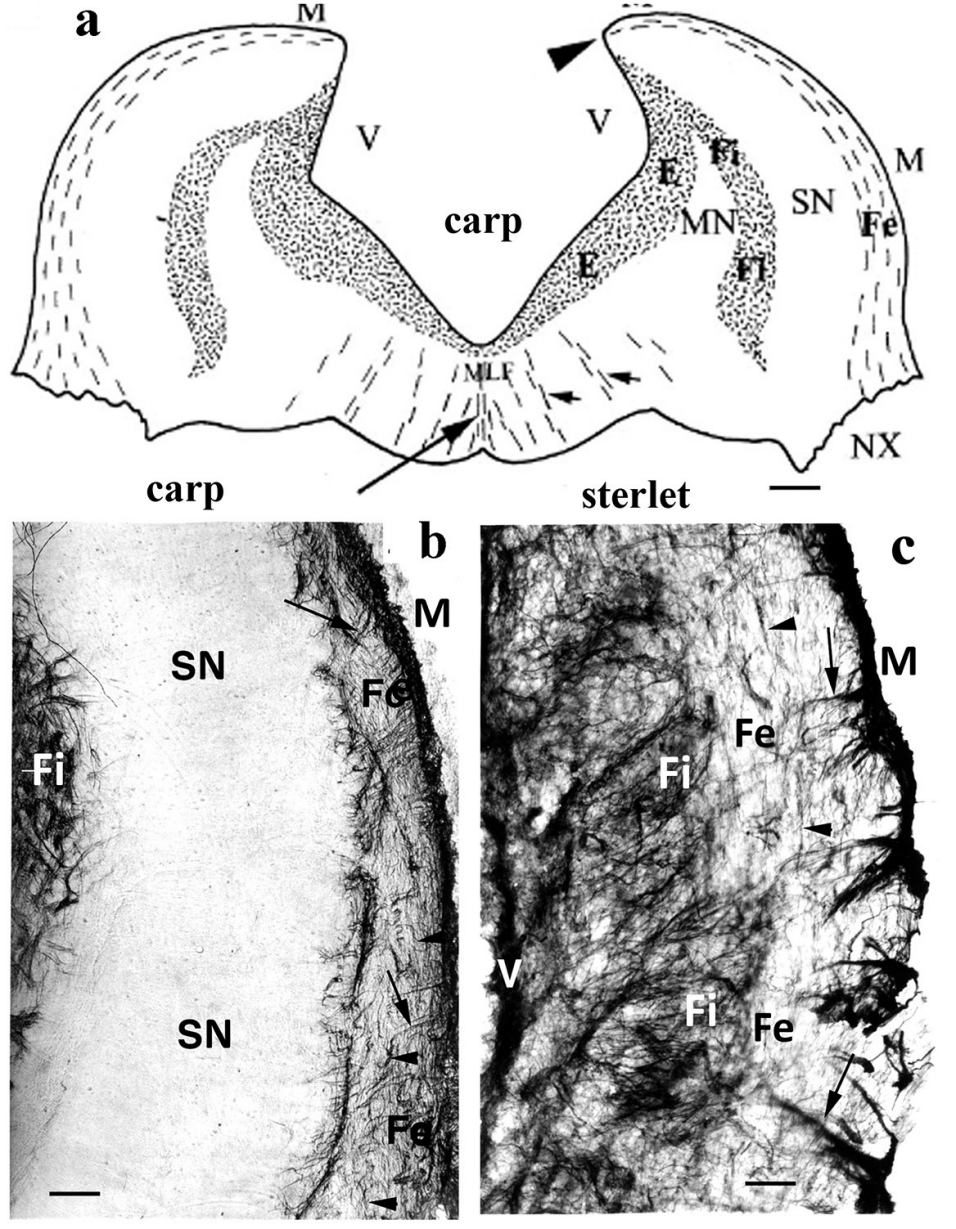
Sterlet and carp, medulla; adapted from Kálmán and Ari (2002). **(a)** Carp; a schematic drawing of a cross-section of the rhombencephalon through the vagal lobes, caudal to the facial lobe. The GFAP-immunopositive areas are stippled. E: ependymal glial plexus; Fe, Fi: external and internal fiber zones; MN, SN: motoric and sensory neurons of the vagal lobe; M: meningeal surface; MLF: medial longitudinal fascicle; V: ventricular surface; arrowheads: the torn attachment of the missing roof of the 4th ventricle; long arrow: midline glial septum; short arrows: smaller glial septa. Scale: 400 μm. **(b)** Carp; a “chemosensory” brain (Kortschall et al., 1998), teleost; the lateral part of the vagal lobe, between the external and internal fiber zones, contains a wide GFAP-free zone of sensory neurons. This layered structure, which outbulges into a “vagus lobe” only exists in the Barbinae and Cyprininae subfamilies. Scale bar: 100 μm. **(c)** Sterlet, also a “chemosensory” but non-teleost fish. Outer and inner fiber zones with looser and denser systems of glial processes (arrows, arrowheads) are found; however, there is no GFAP-free zone between them. Scale bar: 100 μm.

A layered structure (Figure 9) of the vagal lobe was found in a barb (Rubio et al., 1992), carp (Kálmán, 1998; Kálmán et al., 2021), goldfish (Kálmán and Ajtai, 2000; Kálmán et al., 2021), and crucian carp (Kálmán et al., 2021). The zones containing sensory and motoric neurons were GFAP-free. This layered structure seems to be a unique evolutionary acquisition (Nieuwenhuys, 2011). The intense gustatory specialization induced the intense evolution of the vagal, glossopharyngeal, and facial lobes in the Cyprininae and Barbinae subfamilies. These subfamilies of Cyprinidae have “chemosensory” brains according to Kortschall et al. (1998). This layered structure was not found in other fishes, for example, neither in the “chemosensory” but non-teleost sterlet (Kálmán and Ari, 2002) nor in the cyprinid, but not “chemosensory” bram and blake (Table 3; Kálmán et al., 2021).

### 2.6 Cartilaginous fishes

**Figure 10:**
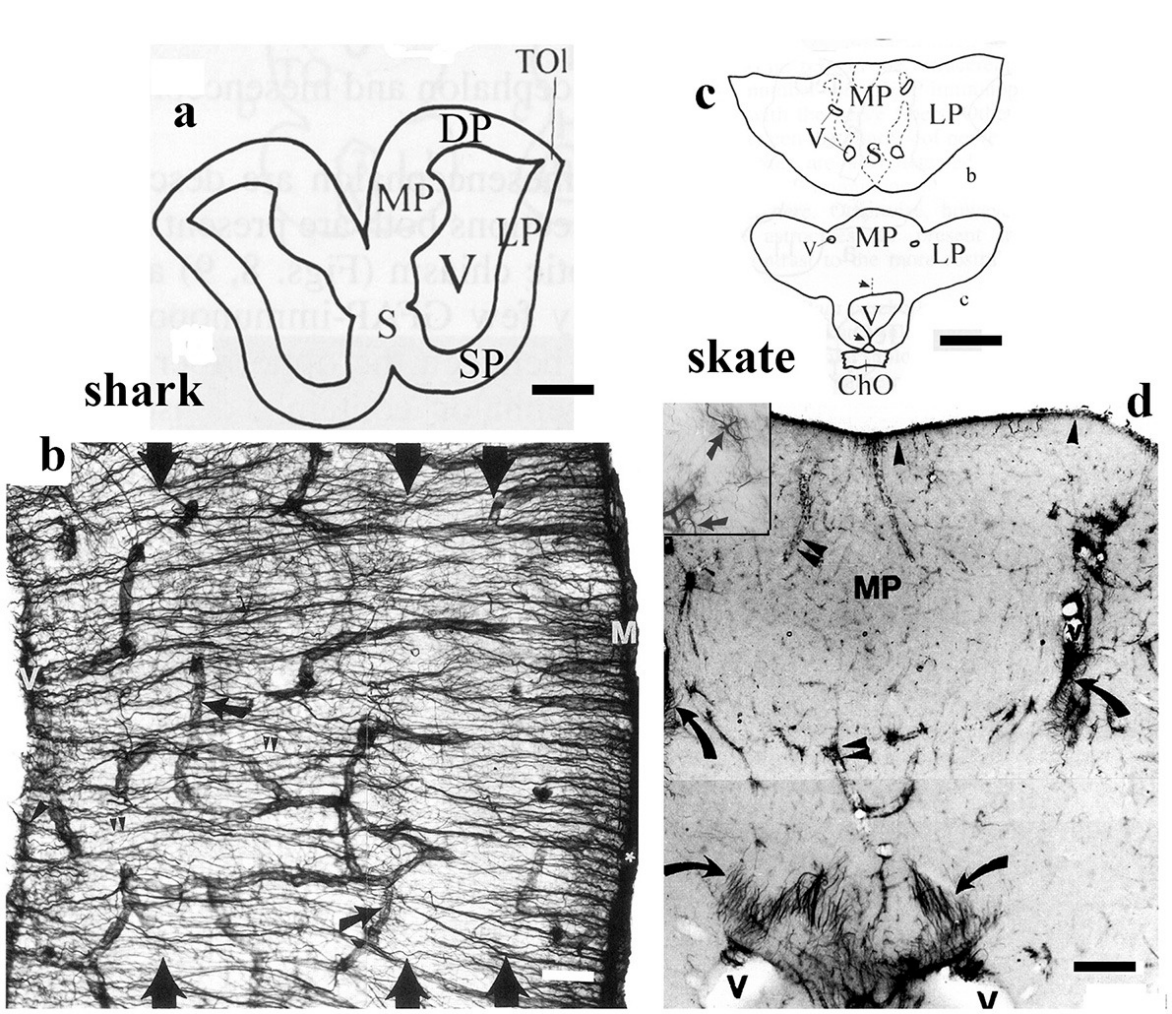
Shark and skate, telencephalic details; adapted from Kálmán and Gould (2001). ChO: optic chiasm; DP, LP, MP: dorsal, lateral, and medial pallia; M: meningeal surface, S: septum; SP: striatum-pallidum; TOl: origin of the olfactory tract, V: lateral ventricle. **(a)** Contour of the telencephalon of a squalomorph shark (spiny dogfish). Scale bar: 2.0 mm. **(b)** Detail of the dogfish brain wall, the GFAP-immunopositive fiber-like tanycytes enmeshing the brain; their main course is from the ventricular surface to the meningeal surface. Arrows point to vessels. There are four layers, but their borders are hardly recognizable (large arrows). Scale bar: 120 μm. **(c)** Contours of the telencephalon of the little skate. Note the thick wall and the narrow ventricles. Scale bar: 1.0 cm. **(d)** Detail of the medial pallium of the little skate. It is poor in GFAP, which is represented mainly by astrocytes (see the numerous dark points, enlarged in the inset). Tanycytes (arrows) are only found around the narrow ventricles. Arrowheads: meningeal surface glia; double arrowheads: vessels. Scale bar: 200 μm.

In batoids (skates and rays, eight species, Table 4), the telencephalon (Figure 10), tectum, and the molecular layer of cerebellum were inferior in GFAP (Kálmán and Gould, 2001; Ari and Kálmán, 2008a), in contrast to the homologous areas of sharks (four species, Table 4). Glutamine synthetase and S-100 protein revealed several astroglial structures in the GFAP-free areas of batoids (Chiba, 2000; Ari and Kálmán, 2008a). The single representative investigated of ratfishes (chimeras, Holocephali) had a glial structure similar to that of sharks (Ari and Kálmán, 2008b).

**Table 4:** Chondrichthyes presented in our studies.

**Superorder**	**Order/ family**	**Species**
**Elasmobranchii- subclass**		
Squalomorpha	Squaliformes/ Squalidae	^*^Spiny dogfish, *Squalus acanthias*, LINNEAUS 1758 (2)
	Pristiophoriformes/ Pristiophoridae	Longnose saw shark, *Pristiophorus cirratus*, LATHAM 1794 (2)
Galeomorpha	Carchariniformes/ Scyliorhinidae	Small-spotted catshark, *Scyliorhinus canicula*, LINNEAUS 1758 (6) Australian swellshark, *Cephaloscyllium laticeps*, DUMERIL 1853 (3)
Batoidei	Myliobatiformes/ Dasyatidae	^**^Japanese red stingray, *Dasyatis akajei*, MÜLLER & HENLE 1841 (4) Common stingray, *Dasyatis pastinaca*, LINNEAUS 1758 (1)
	Myliobatiformes/ Mylobatidae	Bat ray, *Myliobatis californicus*, GILL 1865 (1)
	Torpediniformes/ Torpedinidae	Marbled electric ray, *Torpedo marmorata*, RISSO 1810 (1)
	Rajiformes/ Rajidae	Melbourne skate, *Dipturus whitleyi*, IREDALE 1938 (2) ^*^Little skate, Raia erinacea, MITCHILL 1825 (1) Brown ray, *Raia miraletus*, LINNEAUS 1758 (3) Thornback ray, *Raia clavata*, LINNEAUS 1758 (1)
**Holocephali subclass**		
	Chimaeriformes/ Callorinchidae	^***^Australian ghostshark, *Callorinchus millii*, BORY DE SAINT-VINCENT 1823 (2)

Only taxa demonstrating the main phylogenetic relationships are given based on Compagno (1977) and Winchell et al. (2004).

^*^Kálmán and Gould (2001).

^**^Kálmán et al. (2013).

^***^Ari and Kálmán (2008b), unmarked: Ari and Kálmán (2008a). Numbers in brackets: the number of animals.

### 2.7 Conclusion I

During evolution, a lack of GFAP expression has evolved in some extended brain areas of more advanced groups of vertebrates within different clades, independently of one another. It is an apomorphic phenomenon, a “trend” in brain evolution. The results are summarized in Table 5. The capability of GFAP expression is not lost; it has simply become facultative.

**Table 5:** A summary of correspondence between the positions of the species investigated and GFAP-immunopositivity in some characteristic brain areas.

**Archosauria**			
**Region**	Turtles^*^ **(****Table 2****)**	**Caimans (** **Table 2** **)**	**Birds (** **Table 1** **)**
Pallium dorsale	++	++	Hyperpallium^**^−−
Dorsal ventricular ridge	++	++	Nido–, mesopallium^**^−−
Striatum parvocellulare	++	++	Neotriatum^**^−−
Nucleus rotundus	++	++	−−
Tectum opticum, upper layers	++	++	−−
Bergmann glia	++	++	−−

^*^The position of turtles is unsure (see footnotes at Table 2).

^**^Names after Reiner et al.'s (2004) reform of the terminology of avian neuroanatomy. These regions are homologous with the reptilian ones in the left-side column.

The GFAP-immunonegative areas increased more than the immunopositive ones; therefore, the withdrawal of the latter ones was relative. It appears that these areas are GFAP-immunonegative in mammals and birds, which have undergone enlargement and increased complexity during evolution (see Table 5). These data suggest that the absence of GFAP in certain brain areas may be an evolutionary advantage.

## 3 Discussion: physiological implications of the absence of GFAP

### 3.1 Correlations with other phenomena of brain evolution

#### 3.1.1 Brain size and complexity—“laminar” and “elaborated” brains

According to Butler and Hodos (2005), in each primary vertebrate radiation—cartilaginous fishes, ray-finned fishes, and fringe-finned fishes with tetrapods—two types of brain organization can be distinguished, type I (“laminar”) and type II (“elaborated”). In type I brains, neurons either remain in the periventricular zone or migrate short distances from it. The brain ventricles are large, and the brain wall is relatively thin. In type II brains, neurons migrate extensively; therefore, these brains are larger and composed of numerous nuclei. The ventricles are of reduced size, whereas the brain wall has thickened. All amniotes, teleost fishes, batoids, and the advanced so-called “galeomorph” sharks have type II brains; the others have type I brains (Butler and Hodos, 2005). Note that some batoids exhibit a high range of cerebralization. Their brain weight/body weight ratios overlap the lower range of birds and mammals (Jerison, 1973), mainly due to the evolution of the telencephalon and cerebellum. GFAP-free areas occur only in “elaborated” brains, but not in each of them, see basal teleosts and galeomorph sharks.

#### 3.1.2 Tanycytes and astrocytes

During evolution, two types of astroglia appeared, tanycytes and true, stellate-like astrocytes. The tanycytes (Horstmann, 1954) are thin, elongated, fiber-like glial cells usually of ependymal origin (ependymoglia); they are best visible in Figures 4a, b, 8a, 10b. “Astrocyte,” in the strict sense, refers to stellate-shaped glial cells, which are independent from the ependyma. In contrast, the term “astroglia” comprises both astrocytes and tanycytes, according to the terminology proposed by Mugnaini (1986).

**Table:** Within Aves (Table 2).

**Ranks are based on Jarvis et al. (** **2014** **), Houde et al. (** **2019** **), and Braun and Kimball (** **2021** **)**			
**Region**	**Galloanserae**	**Pigeon, Neoaves, Columbea**	**Neoaves, Passerea**
Entopallium^*^	++	++	−−

^*^In the other regions, the GFAP immunostaining is not different across species investigated.

**Table:** Squamata, Lepidosaurta (Table 2).

**Ranks are based on Wiens et al. (** **2012** **) and Pyron et al. (** **2013** **)**					
**Region**	**Gecko** ^*^	***Timon***	**Snakes**	**Agama** ^**^	**Chameleon** ^**^
Medial pallium	++	+−	+−	−−	−−
Dorsal pallium	++	−−	−−	−−	−−
Lateral pallium	++	−−	−−	−−	−−
DVR	++	+−	+−	−−	−−
Striatum	++	+−	+	+−	−−
Tectum	++	+−	+	+−	−−

^*^Also Lazzari and Franceschini (2005a).

^**^Both species are in the Iguania group.

**Table:** Actinopterygii (Table 3).

**Ranks are based on the study by Betancur-R et al. (****2013**, **2017****)**				
**Region**	**Non-teleostei**	**Basal teleostei** ^*^	**Ostariophysi** ^**^	**Euteleostei** ^**^
Cerebellum, molecular layer	+	+	+−^***^	+
Tectum deep layers	+	+	−−	−−

^*^Basal teleostei: before the separation of Ostariophysi and Euteleostei.

^**^Ostariophysi and Euteleostei are not subsequent but sister groups.

^***^In cyprinids but not in the *Icturus* catfish (Siluriformes, Icturidae).

**Table:** 

**Within Cyprinidae, Ostariophysi (** **Table 3** **)**		
**Region**	**“Non-chemosensitive” brain** ^*^	**“Chemosensitive” brain** ^*^
	**Alburninae, Leuciscinae**	**Cyprininae, Barbinae** ^**^
Lobus X	Relative simple, ++	Multilayered, and there are GFAP layers

^*^According to Kortschall et al. (1998); ^**^Barbinae based on Rubio et al. (1992).

**Table:** Chondrichthyes (Table 4).

**Ranks are based on Compagno (** **1977** **) and Winchell et al. (** **2004** **)**			
**Regions**	**Squalomorpha**	**Galeomorpha**	**Batoids**
Prosencephalon	++	++	+−
Mesencephalon	++	++	−−
Rhombencephalon	++	++	++

++ Confluent, dense GFAP-immunopositive astroglia.

+ Dense, but discontinuous GFAP-immunopositive astroglia.

+− Mainly empty with confined groups of GFAP-immunopositive astroglia.

−− GFAP-immunonegative area, or very scarce GFAP-immunopositive astroglia.

Tables 1–4 present the investigated species and their respective taxonomies.

Regions: listed only those that show characteristic differences.

Mammals are not shown here because there is no other extant synapsid group for comparison.

The appearance of astrocytes, at least their predominance, is phylogenetically younger than that of tanycytes (see also Verkhratsky et al., 2019; Falcone, 2022). In reptiles, ray-finned fishes, and sharks, the predominant GFAP-immunopositive elements are tanycytes. Astrocytes are absent in ray-finned fishes (Kálmán et al., 2021) and turtles (Kálmán et al., 1994, 1997; Lazzari and Franceschini, 2006; Lőrincz and Kálmán, 2020a); they are only complementary elements of astroglia in crocodilians (Kálmán and Pritz, 2001; Lőrincz and Kálmán, 2020b), snakes and lizards (Lőrincz and Kálmán, 2020a), see also Monzón-Mayor et al. (1990), Lazzari and Franceschini (2001, 2005a,b), Ahboucha et al. (2003), and Lőrincz and Kálmán (2020b). The predominance of astrocytes is characteristic of birds and mammals; it appeared independently in these two groups, as it is not seen in either turtles or crocodilians. During their separate evolution, astrocytes also became predominant in skates and rays (Horstmann, 1954; Wasowicz et al., 1999), but – and it is interesting—only in the prosencephalon and mesencephalon, but not in the rhombencephalon (Kálmán and Gould, 2001; Ari and Kálmán, 2008a)—in contrast to mammals and birds, where they dominate the whole brain and spinal cord.

The appearance of astrocytes during evolution can promote the formation of regional differences in the GFAP content. In tanycytes, which are elongated, the scaffold-forming function of GFAP fibrils is mandatory, and a single glial nucleus controls the GFAP expression throughout the full length of the fiber-like cell from the ventricle to the meningeal surface of the brain wall. Once multiple astrocytes have replaced the long tanycytes through the brain wall: “astrocytes demarcate … functional compartments … regulated by single glial cells” (Nedergaard et al., 2003); they can form a versatile glial network, each astrocyte has control over its individual GFAP expression, and the shorter processes may miss the GFAP. Therefore, the GFAP content can be better adapted to the local demands (Mugnaini, 1986**)**, and the unnecessary expression of GFAP can be avoided. Horstmann (1954) and later Reichenbach et al. (1987) attributed the formation of astrocytes instead of tanycytes to the enlargement of brains and the thickening of the brain wall during both evolution and ontogenesis, which is especially conspicuous comparing sharks and rays (Horstmann, 1954) and following mammalian ontogenesis (Reichenbach et al., 1987). Not only astrocyte predominated brain areas are free of GFAP (exceptions are, e.g., euteleost tectum and cyprinid vagal lobe), and not each of these areas is free of GFAP (see, e.g., mammalian and avian pallidum); however, GFAP-free areas extend really only at astrocyte predominance (in mammals, birds, and batoids), and there is no vertebrate brain in which astrocytes predominate but there is no GFAP-free area.

#### 3.1.3 Fibrous and protoplasmic astrocytes

Many of the GFAP-rich astrocytes belong to the “fibrous” type, and forms cordons and frames between and around the axon fascicles of white matter. In contrast, the astrocytes free of GFAP belong to the “protoplasmic” type characteristic of the gray matter (Connor and Berkowitz, 1985; Privat and Ratabul, 1986). Although GFAP is essential for process formation, even GFAP-deficient (GFAP–/–) astrocytes are capable of “stellation” (i.e., process formation) in the presence of neurons (Pekny et al., 1998a). Still, the processes are shorter and finer than those of the GFAP+/+ astrocytes. Although some populations of “protoplasmic” astrocytes do display GFAP immunoreactivity (Connor and Berkowitz, 1985; Walz, 2000), however, in these cells GFAP immunopositivity visualizes only the main branches, so the difference from the fibrous astrocytes is less conspicuous with GFAP immunostaining than following impregnations (Connor and Berkowitz, 1985**)**. One could suppose that the “protoplasmic” type was formed during evolution as the GFAP production has been suspended (become facultative) completely or at least decreased below detectability.

A subdivision of those GFAP-immunonegative cells, which were formerly identified as “smooth protoplasmic astrocytes,” presents NG2 chondroitin sulfate proteoglycan on the surface (Levine and Card, 1987). These cells proved to be oligodendrocyte precursor cells (OPCs) as well, and have been considered a separate type, known as NG2 glia (Nishiyama et al., 1999) or “synantocytes.” Considering their large number persisting beyond the age of oligodendrocyte proliferation, it is likely that these cells also serve other roles (Kimelberg, 2004).

### 3.2 Possible evolutionary advantages

#### 3.2.1 In general

First of all, it is an evolutionary advantage to cease the unnecessary synthesis of a protein. However, it remains unclear whether the saving is significant enough to provide a considerable advantage during natural selection; therefore, other effects may also intoned to be considered. According to an early study by Pekny et al. (1995) in GFAP–/– rats, no abnormalities were observed in their behavior, motility, memory, and BBB function; although the lack of GFAP was not compensated for by the upregulation of other intermediate filament proteins, such as vimentin.

#### 3.2.2 Increased glutamine synthetase activity

The glutamine synthetase activity is more intense in GFAP-free astrocytes. This enzyme neutralizes the toxic ammonium ions by binding them to glutamate (see, e.g., Norenberg and Martinez-Hernandez, 1979; Cooper, 2012). Glutamine levels appear to correlate inversely with GFAP expression. Astrocytes from GFAP–/– mice contained an elevated concentration of glutamine (Pekny et al., 1999a). The GFAP and glutamine synthetase contents are opposite in the “fibrous” and “protoplasmic” astrocytes (Patel et al., 1985; Didier et al., 1986; Ong et al., 1993; Sosunov et al., 2014), although Linser (1985) found glutamine synthetase in fibrous astrocytes as well.

#### 3.2.3 Voltage-gated ion channels

Walz (2000) distinguishes “complex” and “passive astrocytes.” The “complex” ones, which contain no or hardly detectable GFAP, have voltage-gated K(+) and Na(+) channels. These channels can activate or inactivate astrocytes, helping to stabilize the ionic environment of neurons (McNeill et al., 2021). The disadvantageous extracellular K^+^ accumulation evoked by membrane depolarization was lower in the vicinity of GFAP–/– astrocytes than that of GFAP+/+ astrocytes (Anderova et al., 2001). In contrast, “passive” astrocytes, characterized by high GFAP content, lack voltage-gated ion channels (Walz, 2000).

#### 3.2.4 GFAP influences astrocyte effects on neuronal activity

Astrocyte processes contact synapses and may modulate synaptic function, synaptic efficacy, for example, long-term potentiation and depression. Deletion of GFAP increases the former one in the hippocampus (McCall et al., 1996), whereas it decreases the latter one in the cerebellum (Shibuki et al., 1996). Astrocyte processes lacking GFAP are remarkably mobile and therefore have significant effects on neuronal function (Theodosis et al., 2008).

The functional state of several neuron groups changes in parallel with the GFAP content of their astroglia. Retraction of astrocytic processes promotes interaction between neurons and the diffusion of transmitters, whereas expansion reduces neuronal excitability by wedging them apart (Theodosis et al., 2008; Wang and Purpura, 2018). Decrease or increase of GFAP content, redistribution of GFAP to or from the perikarya (Wang et al., 2017), and GFAP depolymerization or polymerization (Wang and Purpura, 2018) are found in the background of the retraction or expansion of processes.

The correlation between the expansion/retraction of astrocytic processes and the activity of the surrounded neurons is well-documented in the supraoptic nucleus in the case of lactation and suckling (Theodosis et al., 2008; Wang and Hatton, 2009; Wang and Hamilton, 2009; Wang et al., 2017), and dehidration/water overload (Wang and Hamilton, 2009; Wang and Purpura, 2018; Wang et al., 2021). Similar correlation was found in the arcuate and preoptic nuclei in the active phases of reproduction (Theodosis et al., 2008; Steinman et al., 2013), in the suprachiasmatic nucleus with the circadian rhythms (Fernandez-Galaz et al., 1999; Theodosis et al., 2008; Lawal et al., 2022), and in canaries in the song center in the period of song learning (Kafitz et al., 1999).

#### 3.2.5 The lack of GFAP can be a secondary phenomenon

However, the causal relations remain to be elucidated: whether the alteration of astrocytes and GFAP staining is a primary phenomenon (Lawal et al., 2022), or only a consequence of the neural processes, a secondary reaction (Theodosis et al., 2008). GFAP expression is modulated by signaling molecules elicited by neuronal activity and hormones (Li et al., 2020). Neuronal activity, an increase in K^+^ or glutamate, may lead to an increase in GFAP fiber formation (Heimfarth et al., 2016; Wang et al., 2021). Afferent activity blockade evoked GFAP production in the chicken cochlear nucleus (Canady and Rubel, 1992). N-methyl-d-aspartate (NMDA) receptors, voltage-dependent calcium channels as well as metabotropic glutamate receptors induce hyperphosphorylation, thereby depolymerizing GFAP (Heimfarth et al., 2016; Wang et al., 2021). The depolymerized form is not detectable with immunohistochemical reaction (Hajós et al., 1993). However, I found no data indicating that neuronal activity in permanently GFAP-free areas (e.g., caudate-putamen complex in rats) surpasses that found in GFAP-rich areas (e.g., the globus pallidus).

#### 3.2.6 Lack of GFAP and vimentin improves post-lesion regeneration

Axon growth and regeneration are held to be inhibited by GFAP-containing glial processes, which appear in the *post-lesion* glial reaction, and demarcate the lesion (Reier, 1986; Hatten et al., 1991; Menet et al., 2001**)**. Inactivation of the GFAP and vimentin genes, the absence of GFAP and vimentin prevents the *post-lesion* reactive hypertrophy of astrocytic processes and improves post-traumatic regeneration (Pekny et al., 1999b; Pekny and Pekna, 2004; Wilhelmsson et al., 2004; Holy and Pekny, 2015). It is worth noting that, in *post-lesion* glial reaction (but not in intact cerebral tissue), vimentin substitutes for GFAP in process formation; therefore, both are to be eliminated. The absence of GFAP and vimentin could induce a reorganization of the actin network, influenceing the composition of the extracellular matrix (ECM) and adhesion molecules, thus yielding an enhanced pearmeability of astrocytes to axon growth (Menet et al., 2001). However, it is usually not considered that the lack of regeneration in the mature central nervous system (CNS) is hardly a “mistake” of evolution. Possibly, adult regeneration could lead to maladaptive responses and mistaken connections (Aswendt et al., 2022). Note that *post-lesion* reactive GFAP expression also occurs in the areas free of GFAP immunoreactivity when they are intact (see, e.g., Figures 3c, d), and considerable regeneration does not occur either.

#### 3.2.7 Lack of GFAP allows more plasticity

The presence or absence of GFAP, which may promote or inhibit, respectively, the synaptic plasticity, the rebuilding and re-arrangement of synapses to adapt to new situations (Missler et al., 1994; Finch, 2003; Wilhelmsson et al., 2004, 2019). The presence of astrocyte processes along the neuronal surface hampers the formation of synapses, whereas their absence allows it (Theodosis et al., 2008).

An increased neuronal differentiation was observed *in vitro* in the presence of GFAP–/–Vim–/– astrocytes, as more neurons survived and escaped apoptosis (Widestrand et al., 2007; Wilhelmsson et al., 2012). However, *in vivo*, in the adult mammalian brain, neither neurogenesis nor axon regeneration occurs in the GFAP-negative areas.

The ECM also influences synaptic plasticity (see, e.g., Song and Dityatev, 2018; McKeon and Silver, 1995). ECM accumulates around certain types of neurons, forming perineuronal nets (PNN, for reviews see Bosiacki et al., 2019; Duncan et al., 2019; Jakovljevic et al., 2021). PNN can be visualized by *Wisteria floribunda* agglutinin (WFA), which binds to N-acetylgalactosamine, a subunit of hyaluronan, the “backbone” of ECM; see, for example, Hilbig et al. (2000). The distribution of WFA has a similar pattern to that of GFAP in rats (Hilbig et al., 2000; Gáti et al., 2010); in other animals, the WFA distribution has not been studied enough.

The cytoskeletal system, including GFAP, is connected with the astrocyte membrane, for example, with the membrane-embedded glutamate-aspartate transporter (GLAST), the ezrin (Sullivan et al., 2007), the dystrophin-dystroglycan complex (Hendriksen et al., 2016; Li et al., 2020), the plectin of the plakin family, and the integrin (α6β4) (Potokar et al., 2020), which may form focal adhesions (Melrose, 2019). Hyaluronan synthetase and CD44 receptor bind the hyaluronan to the cell membrane (Roszkowska et al., 2016; Miyata and Kitagawa, 2017). In this way, GFAP can stabilize the ECM, and through the ECM, it also stabilizes the PNN. A stable PNN inhibits lateral diffusion (Valenzuela et al., 2014) and, therefore, stabilizes synapses and inhibits their plasticity (Bosiacki et al., 2019; Miyata and Kitagawa, 2017; Duncan et al., 2019; Yang, 2020; Jakovljevic et al., 2021). Taking these together, it is to be supposed that the absence of GFAP may promote plasticity and functional adaptation of astrocytes, and therefore that of neurons as well.

### 3.3 The possible adverse effects

#### 3.3.1 Vulnerability of the white matter

GFAP provides the maintenance of astrocyte processes and their resistance to mechanical stress. Fibrous astrocytes form “cordons” along the brain tracts; they are rich in both GFAP and CD44 (Sosunov et al., 2014). The hyaluronectin mesh, which surrounds the myelinated axons, is likely produced by GFAP-producing white matter astrocytes since hyaluronectin and GFAP showed similar distributions (Bignami and Dahl, 1986; Bignami et al., 1992). In this way, GFAP is necessary for the integrity of white matter architecture and the long-term maintenance of myelination (Liedtke et al., 1996; Li et al., 2020). In GFAP-free mice, the vulnerability of white matter increases to traumatic injury, ischemia (Galou et al., 1997; Nawashiro et al., 1998, 2000), and neurotoxicity (Otani et al., 2006).

#### 3.3.2 The reactive gliosis is impaired

In GFAP–/– Vim–/– mice, astrocytes do not form cytoplasmic intermediate filaments, the reactive gliosis is impaired (Pekny et al., 1999b; Menet et al., 2001; Pekny and Pekna, 2004; Wilhelmsson et al., 2004; Holy and Pekny, 2015), and the infarcts developing after ischemic strokes are larger (Li et al., 2008). The advantageous effect for axon regeneration was mentioned in the previous subchapter. However, neither of these effects manifests itself in the GFAP-free brain areas of “wild” animals, where reactive gliosis also develops following lesions. Note that in cultured astrocytes and *post-lesion* glial reaction, vimentin substitutes for GFAP in process formation; but it does not happen in the intact adult cerebral tissue (Pekny et al., 1999b; Menet et al., 2001).

#### 3.3.3 The blood-brain barrier may be compromised

According to Ding et al. (1998), Nawashiro et al. (2000), and Jurga et al. (2021), GFAP is necessary in astrocytes to help endothelial cells in the formation of the BBB. Although Pekny et al. (1995) found no extravasation of Evans blue in GFAP knockout mice, which implied the existence of an intact BBB, in a later study, however, they discovered that GFAP-positive astrocytes are necessary to restrict the passage for some hydrophilic molecules, for example, saccharose (Pekny et al., 1998b). According to our experience, perivascular astrocytes usually display GFAP immunopositivity, even in areas that are otherwise devoid of it (see, e.g., Figures 2c, 3b).

#### 3.3.4 Decrease of the resistance to osmotic changes

Extracellular water content, osmotic pressure, and sodium/potassium concentration are balanced by astrocytes (Anderova et al., 2001). GFAP is a key component in the cytoskeletal network implicated in cell volume regulation. Cytoskeleton makes astrocytes resistant to cell swelling in response to hypoosmotic stimulation (Ding et al., 1998; Wang et al., 2021), its impair increases their susceptibility to cytotoxic edema (Ding et al., 1998; Nawashiro et al., 2000; Anderova et al., 2001; Bélanger et al., 2002; Butterworth, 2010); its lack could facilitate cell swelling, and decrease the compensatory efflux or influx of ions and organic osmo-equivalents, mainly taurine (Ding et al., 1998), The interactions between GFAP and F-actin networks and membrane anchor proteins, for example, plectin, dystrophin squeezes water out and reduces volume (Wang and Hatton, 2009). The expression of AQP4 water-pore protein is in parallel with the levels and spatial distribution of GFAP (Wang and Hatton, 2009).

#### 3.3.5 The defension from glutamate excitotoxicity is decreased

Astrocytes take up the excess of glutamate produced by neuronal activity (synaptic release), protecting the neurons against glutamate excitotoxicity. GFAP knockout mice exhibit a reduced glutamate clearance due to a decrease in glutamate transporters (Hughes et al., 2004; Sullivan et al., 2007; Butterworth, 2010; Kim et al., 2018). Glutamate is also the source of gamma-aminobutyric acid (GABA), which is produced by astrocytes and serves as a neurotransmitter for neurons (“GABA shunt,” Madsen et al., 2010).

#### 3.3.6 Other processes

The elimination of free oxidative radicals, which are formed during oxidative metabolism, is impaired. It appears that GFAP influences this process, although probably indirectly, by mediating other factors; the mechanisms involved remain to be determined (De Pablo et al., 2013).

Vesicle and enzyme trafficking are also impaired, as well as gliotransmitter release, since GFAP, as a component of the cytoskeletal system, has some role in transport functions. Several enzymes are associated with GFAP, e.g. vesicular GABA transporter (Potokar et al., 2007; Li et al., 2020; Wang et al., 2021). GFAP serves as a platform for interactions between different signals, and between enzymes and their substrates.

Elevated factors of plasticity may also render them vulnerable to abnormal structural changes, as seen in psychiatric diseases (García-Cabezas et al., 2017). Decreased GFAP was found in association with schizophrenia models (Kim et al., 2018; Li et al., 2020).

### 3.4 Conclusion II

What was the role of the relative “withdrawal” of GFAP expression in brain evolution? It cannot be answered yet, definitely! The most probable candidates are plasticity, better adaptability to neuronal activity, and the absence of unnecessary protein synthesis. However, there are functions that depend on the presence of GFAP. The balance of these antagonistic consequences determines that in a given area, astroglia express GFAP permanently or only upon necessity, for example, following injury. Comparative studies on brain areas that are rich in GFAP in one species but poor in another (e.g., entopallium) may promote the understanding of the role of GFAP in neural networks.

However, one must take into consideration that

a) It is possible that the “withdrawal” of GFAP is not a primary phenomenon but a consequence of the alterations of neural networks during evolution and
b) Most of the experimental data on the lack of GFAP are from genetically manipulated animals or cell cultures made incapable of GFAP production, whereas the GFAP-immunonegative astrocytes of “wild” animals are capable of it in necessity, for example, following a lesion.

## Data Availability

The original contributions presented in the study are included in the article/supplementary material, further inquiries can be directed to the corresponding author.
